# New Trends in Instrumentation and Complex Techniques in Spine Surgery

**DOI:** 10.1155/2015/216384

**Published:** 2015-12-30

**Authors:** Alessandro Landi, Roberto Delfini, Alessandro Ricci, Andrea Barbanera, Giulio Anichini, Christian Brogna

**Affiliations:** ^1^Department of Neurology and Psychiatry, Neurosurgery, “Sapienza” University of Rome, 00181 Rome, Italy; ^2^Department of Neurosurgery, University of L'Aquila, 67100 L'Aquila, Italy; ^3^Department of Neurosurgery, Civil Hospital of Alessandria, 15121 Alessandria, Italy; ^4^Department of Neuroscience, Neurosurgery, Imperial College, Charing Cross Hospital, London SW72AZ, UK; ^5^Department of Neurosurgery, King's College London, London WC2R 2LS, UK

The overall characteristics of the vertebral column are, namely, elastic resistance to movement, twisting potential, and elastic resistance to load bearing. These aspects reflect the three main functional characteristics of spine: motility in all the spatial planes, passive and active resistance to the axial load, and elastic resistance to excessive degrees of movement. In light of this, we can assert that motility at the level of a single metamere should be interpreted not only merely as movement on the three planes but also, and above all, as elastic resistance to dynamic stress on these three planes. In fact, metameric movement depends on an active motility, involving the intervertebral disc, the articular masses, and the muscular structures, and a passive motility, involving the disc, ligamentous system, and articular masses. In light of this, the aim of spine surgery is to decompress the neural structures and neutralize excessive movements while preserving as much as possible the physiological biomechanical properties of the metamere involved. Those objectives are mandatory for every type of pathology in which the spine is involved, such as degenerative, traumatic, malformative, and oncologic ones. In light of technical evolution of surgical instruments and software and of recent introduction of new surgical approaches, the future of spinal surgery is changing. The articles contained in the present issue include both reviews and original case-based studies focused on innovative technologies, new surgical techniques, and approaches to the spinal pathology, with the aim of describing experiences, tips and tricks, and lessons learnt.

The need to preserve, as much as possible, the biomechanical characteristics of the spine has become impelling, considering the long term results of the traditional surgery. It has become evident that surgery is effective in symptoms control in the short and medium term, but in the long term it might lead to physiological and biomechanical complications, if the specific spinal anatomical and functional features are not preserved. Considering those results, the research and the technological development in spinal surgery are the main characters of important innovations capable of assisting the work of the surgeon and the wellbeing of the patient. Such innovations are being created in three main specific fields.

*Development of New Surgical Techniques.* The main aim is to make spine surgery less invasive and safer for the patient, focusing on the reduction of hospitalizing times, the reduction of procedure related risks, and the accelerating of functional recovery. Goals of the new techniques are both the decompressive and the reconstructive phase, generally performed as fusion. The percutaneous MIS and the endoscopic surgery have become always more important in spine surgery. They allow performing traditionally open procedures, such as microdiscectomy or spinal fusion, through percutaneous and endoscopic approaches extremely minimally invasive and atraumatic for the tissues. Moreover the association of minimally invasive techniques with the operative microscope allows the surgeon to minimize the tissue damage gaining optimal results in terms of outcome [1, 2].

Among the most recent stabilization techniques that are worthy to be mentioned are the “cortical bone trajectory screws.” They allow the contextual execution of a stabilization with the insertion of isthmic screws and the decompression through a minimal approach (only few centimeters) [3, 4].

The interarticular stabilization with Facet-Wedge has a role in spinal surgery too. This is a recently developed technique that allows, through the surgical ankyloses of the articular masses, a high strength stabilization [5]. MIS and percutaneous techniques are currently considered the gold standard for the treatment of degenerative, traumatic, and tumoral pathology. Percutaneous pedicle screw fixation in spinal trauma allows a faster functional recovery without requiring external orthesis [6–8]. In tumour surgery, the percutaneous stabilization of primary or metastatic spine tumours allows the patient to undergo adjuvant treatments, such as radiotherapy, in shorter times, with a better improvement in quality of life if compared to the classic standard techniques [7].

The development of new surgical approaches to the spine, such as the lateral approaches XLIF and LLIF [9–12], allows the execution of newer interbody fusion procedures, with a higher rate of fusion. Furthermore, the expansion of motion preservation surgery is revolutionizing the traditional surgical approaches for rigid stabilization and fusion. The creation of dynamic stabilization systems and of disc prosthesis represents the future of spinal surgery, even if the current state of the art needs a further implementation in both technique and materials [13].

*Development of New Technologies.* The main aim is to give the surgeon new technologies, new materials, and new devices capable of the following:

1. Giving help during surgery: increasing safety, precision, and reliability of the procedure, focusing on pedicle screw placement (the rate of revision surgery to rectify misplaced screws ranges from 1 to 5%; the additional cost of one revision surgery to correct a misplaced screw ranges from $17,650 to $27,677). There are several technologies available for this function. “Procedurally integrated neuromonitoring” is able to check in every moment and in real time the functions of the neuromuscular structures during pedicle screw placement or other surgical procedures [14]. “Spinal Neuronavigation” plays a very important role in many spine surgery centres, thanks to the possibility of preoperatively planning the whole surgical procedure and making intraoperative changes through a digital elaboration [15, 16]. Very interesting are the “3D Printed Tubular Guides,” patient matched guides for pedicle screw placement that are built by a 3D printer on the basis of a preoperative CT scan. This custom-made solution, as also Neuronavigation, provides a more precise and accurate screw insertion, particularly in patients with deformity and alteration of the normal surgical anatomy, and a correct sizing of the screws, reducing the risk of pull-out [16]. The most recently developed device is the “3D Printed Vertebra,” a 3D print titanium customized implant for the substitution of one or more vertebral bodies with a prosthesis designed on the patient [17].
2. Reducing the exposure to ionizing radiations for both patient and surgical staff: worth mentioning are the new “robot based imaging and 3D fluoroscopy” systems, able to perform high quality 3D reconstructions with a robotic C-arm [18]. It can be integrated with the neuronavigation system, with a reduction in the ionizing radiation exposure. Another robotic system developed in spinal surgery is the “Robotic Arm Guidance.” This system, based on a preoperative planning developed in a virtual 3D setting, offers a high accuracy in pedicle screw insertion, with a margin of error of 1,5 mm, and can be used in both open and percutaneous procedures [18].

*Development of New Materials.* The research in the field of new biomaterials is fundamental because implant surgery is the basis for the treatment of many spinal pathologies. The development of materials with a high biocompatibility, with biomechanical characteristics similar to the native tissue and capable of promoting tissue regeneration, is opening new and interesting scenarios [19].

From this point of view, the research for new haemostatic materials is gaining good results, with the development of materials to reduce and control the difficult intraoperative bleedings, often related to postoperative consequences for the patient [20].

Moreover, the efforts to find new osteoinductive materials, such as nanomolecular hydroxyapatite, might result in a faster and more physiological ossification, respecting the biomechanical characteristics and reducing time of hospitalization and related expense [21].

*Conclusions.* Novel technologies actually are developing to help the surgeon to perform a most accurate, safe, and adequately planned surgery and to reduce the exposure to ionizing radiations. Instead new techniques are developing as an alternative to standard surgical approaches with specific surgical indications, with the aim of reducing tissue damage, length of hospitalization, and postoperative pain, and of promoting a faster functional restoration. New trends in spinal surgery are going towards a customization of the implants, tailored to the single patient, and towards minimally invasive, percutaneous, and endoscopic surgery. Unfortunately behind every new technology and technique there is a constant pressure of the companies. Clearly, in light of this, any of them can be validated only by experience, follow-up, and an accurate risk-benefit ratio. We hope that this special issue would shed light on major innovative trends and complex techniques in spinal surgery and attract attention by the scientific community to pursue further investigations leading to the rapid implementation of these innovations in the spinal fields.
